# Modeling the oxidative consumption of curcumin from controlled released poly(beta-amino ester) microparticles in the presence of a free radical generating system

**DOI:** 10.1093/rb/rbz002

**Published:** 2019-02-13

**Authors:** Carolyn T Jordan, J Zach Hilt, Thomas D Dziubla

**Affiliations:** Department of Chemical and Materials Engineering, University of Kentucky, Lexington, KY, USA

**Keywords:** oxidative stress, wound healing, oral mucositis, drug delivery

## Abstract

Despite the promise of its therapeutic benefits, curcumin as a free molecule has failed to demonstrate significant clinical success. Arguably, its inherently poor stability and rapid clearance is a significant reason for these negative outcomes. The incorporation of curcumin into the backbone of a crosslinked hydrogel that utilizes poly(beta-amino ester) (PBAE) chemistry can provide a tunable protective network with the ability to release at a controlled rate while improving its therapeutic potential. Kinetics of curcumin conjugated PBAE microparticles controlled release delivery system in the presence of oxidative environments was studied for the first time, where consumption rates of active curcumin and release products were obtained. The constituent amount of curcumin present in solution was improved by incorporating the active into the network in comparison to curcumin as a free drug. Modeling curcumin conjugated PBAE microparticles will provide a design platform to improve translation and overall success in delivering a therapeutic agent that matches levels of oxidative stress.

## Introduction

The cytosolic balance between reactive oxygen species and antioxidants is essential to the physiological health of a cell [1]. However, under a variety of pathophysiological conditions (e.g. radiation injury [2], inflammation [3], acute lung injury [4]), cells are no longer able to maintain this balance, resulting in an accumulation of free radicals, defined as oxidative stress [5].

Oral mucositis (OM), an adverse side effect of head and neck cancer radiotherapy, is one of many specific examples of the potentially debilitating effect that oxidative stress can induce [6]. The exposure of ionizing radiation to healthy epithelial tissue initiates breaks in the DNA and inhibits rapid replication of cells in the submucosa. Water molecules found within the cell can also interact with electrons and upon interaction, form hydroxyl radicals [2, 7, 8]. This interferes with the intracellular regulation, leading to the formation of superoxide anions. These free radicals are linked to a specific inflammasome, nucleotide-binding domain and leucine-rich repeat containing Proteins 1 (NRLP1) that activates pro-inflammatory cytokines, such as interleukin 1β (IL-1β) [9]. These secondary messengers transmit signals to stimulate pro-inflammatory markers such a nuclear factor-kappa B, and activator proteins, such as activator protein-1 [10]. The activation and upregulation of these receptors activate matrix metalloproteinases, which can cause tissue damage and lead to apoptosis and cell death [11]. These detrimental cell signaling cascades continue to inhibit proliferation of healthy epithelial cells, which ultimately lead to the formation of severe ulcers within the oral cavity [8]. Despite the prevalence of this disorder, there exist few treatments other than palifermin, a keratinocyte growth factor. Although palifermin has been shown to reduce onset and severity of OM, it is a therapeutic limited to patients with hematologic malignancies [12]. Temporary treatments such as topical anesthetics and analgesics are implemented during prognosis [6]; however, a successful drug to scavenge free radicals and maintain homeostasis to reduce the onset of OM for all patients does not exist and is in high demand.

Curcumin, a potent antioxidant, has been a promising candidate for the control of inflammation and serves as a free radical scavenger [13]. However, up to now, no double-blinded, placebo controlled clinical trial of curcumin has shown a positive benefit [14]. Due to curcumin’s hydrophobicity and severe sensitivity to oxygen and light, a controlled delivery system to release curcumin successfully as a therapeutic agent in oxidative stress-induced diseases is of critical need. Recent literature, such as Pauli *et al*., expresses concern on the use of curcumin’s high promise, yet consistent failure as a successful nutraceutical with the countless failures in translational attempts from lab bench to person [14]. Treatments using excessive amounts of free curcumin have been reported to obtain minimal absorption in serum levels, which not only highlights the failure as a free molecule but risks toxicity, due to high localized concentrations of antioxidant at the site of delivery [15]. The ability to understand the interaction between a delivered antioxidant and a free radical promotes the design of a mathematical kinetic rate model that describes the rate of consumption and radical scavenging ability. Design by mathematical analysis creates high promise in the development of a delivery system that will be consistently successful in treatment.

An innovative delivery system, curcumin conjugated poly(beta-amino ester) (PBAE) polymer networks, has been developed previously in the Dziubla and Hilt laboratories to enhance curcumin’s stability, solubility and resistance to aggregation—all problematic attributes presented with curcumin as a free drug [16]. The incorporation of curcumin into the backbone of a hydrolytically degradable crosslinked hydrogel can eliminate the concern of curcumin’s pitfalls and allow for a tunable network with high drug loading capacity that also provides protection to the agent until released. The synthesis, swelling and degradation properties in controlled temperature phosphate buffered saline (PBS) environments [17], as well as the dynamic mechanical analysis of different network compositions [16] have been investigated extensively. These degradable hydrogels can be prepared in a multitude of forms (e.g. films, microparticles (MPs) and nanoparticles). They have successfully prolonged suppression of oxidative stress in cells [17] as well as inhibited H_2_O_2_-induced mitochondrial oxidative stress [18].

Controlled release systems are beneficial in that they are able to provide a constant therapeutic concentration of drug in a system for a controlled period of time [19]. Specific for antioxidant delivery, knowledge of consumption rates of active curcumin released from MP systems can provide insight in dosing and treatment regimens to unique oxidative environments to maintain a regulated environment [20]. Kinetic modeling can be used to model curcumin conjugated PBAE MPs to verify the consumption rates of active curcumin and degradation products in the presence of free radicals. We hypothesize that curcumin released from MPs of different compositions will be consumed based on the concentration of free radicals present and rate of drug release.

In this work, we report on the consumption of curcumin as a free molecule and the degradation product release and consumption profile from three controlled release curcumin conjugated PBAE MP systems. This will be the first time PBAE MP release profiles will be characterized in the presence of an oxidizing agent. Two different concentrations of 2, 2-azobis(2-amidinopropane) dihydrochloride (AAPH) were evaluated *in vitro* as the oxidative environment due to its ability to degrade into alkyl radicals when introduced to temperatures of 37°C.

Studies quantifying curcumin degradation in an oxidative environment were utilized to develop a set of mathematical expressions to describe the oxidative consumption rates of both curcumin as a free molecule and curcumin and its residual degradation product, curcumin monoacrylate, after they have been released from the PBAE crosslinked network. The consumption rate constants can be used to model theoretical curcumin levels over time and the change in concentration of reactive oxygen species in an environment. The model also demonstrates the novelty of controlled release versus single dose delivery methods and the effect both delivery methods have on the theoretical baseline levels of reactive oxygen species that are necessary in a regulated cellular environment.

## Materials and methods

### Materials

Curcumin was purchased from Chem-Impex International, Inc. (Wood Dale, IL). 4,7,10-trioxa-1,13-tridecanediamine (TTD), Tween 80 and AAPH, triethylamine and acryloyl chloride were purchased from Sigma Aldrich (St Louis). Poly(ethylene glycol) diacrylate, MW 400 (PEG(400)DA) was obtained from Polysciences Inc. (Philadelphia, PA). Dichloromethane (DCM), tetrahydrofuran (THF), dimethylsulfoxide (DMSO) and acetonitrile (ACN) were purchased from Pharmco-Aaper (Brookfield, CT). No additional purification steps were conducted after materials were received.

### Curcumin conjugated PBAE MP synthesis

Curcumin multiacrylate (CMA) was prepared using an acid chloride-alcohol esterification reaction, as described in Patil *et al*. [21]. Briefly, curcumin and acryloyl chloride were reacted in a 1:3 molar ratio in anhydrous THF in the presence of triethylamine for 24 h in the dark to produce CMA comprised of 45% curcumin diacrylate, 55% curcumin triacrylate and 0.9% curcumin monoacrylate characterized by reverse-phase high performance liquid chromatography (HPLC) (Water Phenomenex C18 column, 5 µm, 250 mm (length) × 4.6 mm (ID) on a Shimadzu Prominence LC-20 AB HPLC system) coupled with a UV-Visible (UV-Vis) detector set at 420 nm. The method used was a 20-min water/ACN gradient supplemented with 0.1% (v/v) phosphoric acid at 1 ml/min that started at 60% water/40% ACN to 0% water/98% ACN over 12 min, remained isocratic for 3 min, and then equilibrated back to 60% water/40% ACN for the remaining 5 min.

PBAE films were synthesized as previously stated in Patil *et al*. [16]. CMA and PEG(400)DA were reacted with a diamine crosslinker, TTD, at a ratio of total acrylates to amine protons of 1.0 (RTAAP = 1.0) with two different compositions (26 wt% curcumin and 32 wt% curcumin loading). Upon Michael addition, crosslinked PBAE bonds were formed. 1.5 times the total monomer mass of the film of anhydrous DCM was used as the reaction medium. Curcumin was dissolved in half of the anhydrous DCM. PEG(400)DA was added to a separate centrifuge tube with the remaining amount of DCM. TTD was added to the PEG(400)DA/DCM solution and vortexed immediately. After 5 min at room temperature, the PEG(400)DA/TTD solution was vortexed while CMA was added dropwise quickly and the pre-polymer solution was immediately poured into casting ring on an aluminum covered glass plate and left at room temperature for 1 h. The film was then transferred to a 50°C convection oven for 24 h to complete the reaction and evaporate excess solvent.

Crosslinked films of 0.4 mm thickness were washed for 1 h at 40 ml per 1 g of polymer with anhydrous ACN (five times) to remove any unreacted monomers. Films were placed under vacuum at 50°C overnight to complete drying. MPs were obtained by cutting the film into small pieces and placed into a milling tube with 1 wt% magnesium stearate as a lubricant. Using a 6775 Freezer/Mill Cryogenic Grinder, the film was milled for 10 min at 15 cycles per second, with a pre- and post-cool setting of 3 min. MPs were left on the bench top until the tube reached room temperature to prevent any moisture from condensing into tube. MPs were then collected and dried overnight on a lyophilizer to remove any excess moisture and stored at −20°C in a sealed bag with desiccator pack until future use.

### Free curcumin stability and consumption in the presence of AAPH

Curcumin was dissolved at 80 mg/ml in anhydrous DMSO and 6.25 µl was added under constant vortexing to a 10 ml 0.1% (w/v) Tween 80 PBS (pH 7.4) solution to reach a final concentration of 50 µg/ml curcumin. Periodic samples were collected and read at 420 nm using a Cary 50 UV-Vis Microplate Reader to monitor absorbance of solution over 24 h. Curcumin of the same concentration was also introduced to a 10 mM AAPH and 100 mM AAPH 0.1% (w/v) Tween 80 PBS (pH 7.4) solution and periodic samples were collected and read directly at 420 nm.

### MP degradation profiles in the presence of AAPH

Microparticle systems were degraded in a 0.1% (w/v) Tween 80 PBS (pH 7.4) solution, 10 mM AAPH/0.1% (w/v) Tween 80 PBS (pH 7.4) or a 100 mM AAPH/0.1% (w/v) Tween 80 PBS (pH 7.4) solution over 24 h at the same theoretical final release concentration of curcumin. One millilitre samples of the supernatant of the control were collected every 2 h and analyzed by the UV-Vis microplate reader. The volume was replenished after each time point. Independent samples for each time point were prepared for the MP release in AAPH solution to maintain a consistent ratio of free radicals to curcumin throughout the 24-h study.

### Microparticle degradation product consumption in the presence of AAPH

Microparticles were fully degraded in a 0.1% (w/v) Tween 80 PBS solution (pH 7.4). AAPH was added to the degradation products to a concentration of either 10 or 100 mM and analyzed directly over time using HPLC coupled with UV-Vis to analyze curcumin and residual curcumin acrylated release products over time in the presence of AAPH using the same method described for CMA analysis.

### Model development

#### Oxidative consumption rate of curcumin

Based on the experimental data collected regarding the consumption profiles of curcumin as a free molecule, a kinetic rate model was developed to describe the oxidative consumption of curcumin. Observing the interactions between three main components in solution (AAPH, free radicals produced and curcumin) using a first principles approach, a set of rate equations were established. It was assumed that curcumin consumption was solely dependent on the interaction with the radical formed by thermal decomposition of AAPH.

In an AAPH solution, the compound can thermally decompose into either alkyl radical or peroxyl radicals in the presence of molecular oxygen:

(1) $$\mathrm{AAPH}\;\overset{\Delta }{\to }\;2R\cdot \;\overset{O_{2}}{\to }\;2R\mathrm{OO}\cdot$$

It is assumed that radicals are generated upon thermal decomposition of AAPH. As such, a first order rate was assumed (*k*_A_ = 1.26 × 10^−6^ min^−1^) based on Werber *et al*. [22], and can be expressed in the following equation:

(2) $$\frac{dC_{A}}{\mathrm{dt}}=-k_{A}C_{A}\;$$

where *C*_A_ is the concentration of AAPH. The thermal decomposition of AAPH produces two molecules of radicals for every one molecule of AAPH. Based on the instability of general alkyl/peroxyl radicals, the fast half-life and elimination of the free radicals produced are on the order of 1 ms [23], which is assumed to be first order (*k*_el_ = 4.16 × 10^4^ min^−1^). In an AAPH solution, the concentration of free radicals present can be simplified as:

(3) $$\frac{dC_{R}}{\mathrm{dt}}=2k_{A}C_{R}-k_{\mathrm{el}}C_{R}\;$$

where *C*_R_ is the concentration of free radicals. The change in curcumin concentration is dependent on both the concentration of curcumin and free radicals present over time:

(4) $$\frac{dC_{C}}{\mathrm{dt}}=-k_{C}C_{C}C_{R}\;$$

where *C*_C_ is the concentration of curcumin in solution. In the presence of curcumin, Equation (3) can be modified to incorporate the dependence of free radical concentration on the interaction with curcumin present.

(3a) $$\frac{dC_{R}}{\mathrm{dt}}=2k_{A}C_{R}-k_{\mathrm{el}}C_{R}-k_{C}C_{C}C_{R}\;$$

#### Oxidative consumption rates of curcumin and curcumin monoacrylate released from the MP network

To better model the consumption rate parameters of the released curcumin (*k*_c_) and curcumin monoacrylate (*k*_CM_) from MPs, an additional equation, dependent on the free radical concentration, was added to describe the change in concentration of curcumin monoacrylate in the system over time:

(5) $$\frac{dC_{\mathrm{CM}}}{\mathrm{dt}}=-k_{\mathrm{CM}}C_{R}C_{\mathrm{CM}}\;$$

The free radical concentration is then also dependent on the concentration of curcumin monoacrylate modifying Equation (3a) to the following:

(3b) $$\frac{dC_{R}}{\mathrm{dt}}=2k_{A}C_{R}-k_{\mathrm{el}}C_{R}-k_{C}C_{C}C_{R}-k_{\mathrm{CM}}C_{\mathrm{CM}}C_{R}\;$$

The set of mathematical expressions for the free molecule and degradation products were solved simultaneously using ODE15s in MATLAB while the unknown consumption rate parameters were minimized over the experimental data by sum of squared errors (fminsearch). These parameters were then used to find consumption rate parameters of curcumin monoacrylate found within the solution from the fully degraded MP system and the correlation factor was calculated to evaluate the degree of fit.

#### Demonstration of controlled release antioxidant delivery

Six zero order rates of curcumin release were investigated and compared with a one-time bolus of the equivalent amount of curcumin over 1440 min (Fig. 7). The specified rate was added to the curcumin concentration Equation (4) as follows:

(4a) $$\frac{dC_{C}}{\mathrm{dt}}=\mathrm{specified}\;\mathrm{rate}-k_{C}C_{C}C_{R}\;$$

**Figure 1:**
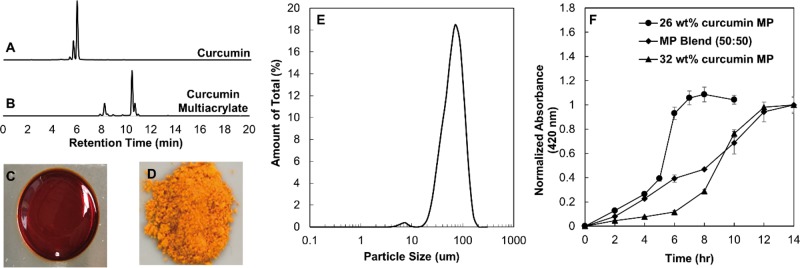
Characterization of curcumin conjugated PBAE MP. (**A**) PBAE film. (**B**) Cryomilled curcumin conjugated PBAE MPs. (**C**) Distribution of MP size. (**D**) Degradation profiles of 26, 32 wt% and 50:50 blend of 26 and 32 wt% curcumin MP. (mean ± SEM, *n* = 3).

For one-time delivery treatments, the initial concentration of AAPH was kept constant at 10 mM and the only information modified in the model was the initial concentration of curcumin present.

## Results and discussion

### Curcumin conjugated PBAE MP characterization

Curcumin, characterized by HPLC (Fig. 1A) was successfully modified to a multiacrylate co-monomer (Fig. 1B) to be incorporated into the PBAE network. The shift in retention time is due to the addition of acrylate groups to the molecule. The peaks were compared with Patil *et al*. [21] to identify the monoacrylate, diacrylate and triacrylate retention times and percentages. Poly(curcumin) films (Fig. 1C) were successfully synthesized and cryomilled (Fig. 1D). Size distribution of the MPs was characterized using a Size Analyzer (Shimadzu SALD-7101) and ranged from 25 to 100 µm (Fig. 1E). Degradation studies of the two systems individually and blended at a 50:50 ratio was conducted in a 0.1% (w/v) Tween 80 PBS (pH 7.4) solution to quantify the cumulative release of curcumin over time. Curcumin release was monitored using UV-Vis spectroscopy at curcumin’s maximum absorbance wavelength of 420 nm. Upon introduction of PBAE MPs to a 0.1% (w/v) Tween 80 PBS (pH 7.4) solution, base-catalyzed hydrolysis occurs and the ester bond is cleaved by a nucleophilic attack from a water group present; however, the rate of hydrolysis is dependent on the degree of hydrophobicity of the polymer backbone. As hydrophobicity increases, the rate of release decreases, slowing the curcumin release rate. The 26 wt% loaded curcumin MPs, with a higher incorporation of the hydrophilic co-monomer, PEG(400)DA, released curcumin over 8 h until the absorbance values plateaued. When incorporating a higher amount of CMA in to the backbone of the network, the rate of release is extended over a 12-h period. For each bulk film system, there is a point at which the integrity of the film is lost, and a burst release can be observed. Due to the increase in surface area of the MP system, the burst release phenomenon is minimized and the release profile appears more linear compared with bulk film degradation. To form a constant release, the 26 and 32 wt% loaded curcumin MPs were blended at a 50:50 ratio. By incorporating the 26 wt% MP system and the 32 wt% MP system, the faster release took place initially and the slower rate of release dominated after 6 h, taking on a constant rate of release with an *R*^2^ = 0.98 when comparing to a constant slope trendline (Fig. 1F).

### Consumption profiles of curcumin

**Figure 2:**
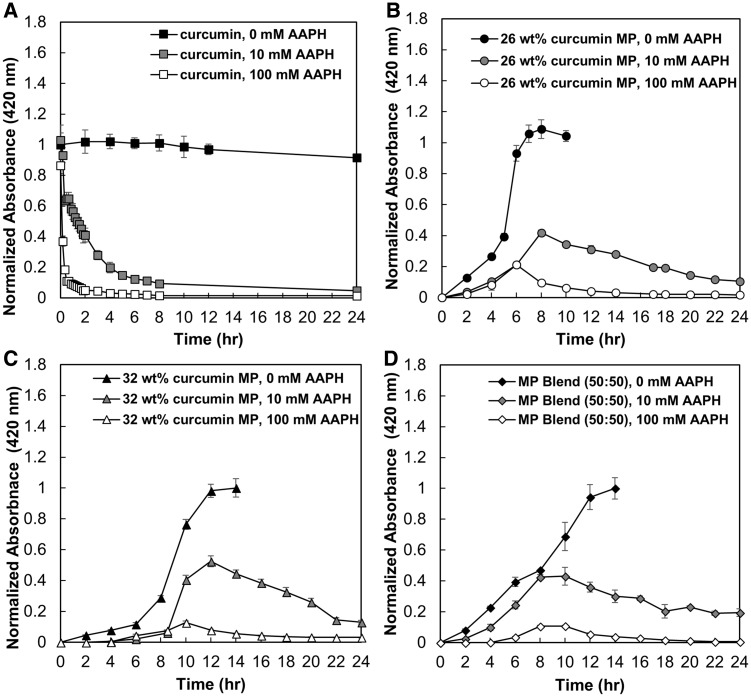
Release and consumption profiles of curcumin and curcumin MPs. (**A**) Curcumin, (**B**) 26 wt% curcumin-loaded PBAE MP, (**C**) 32 wt% curcumin-loaded PBAE MP and (**D**) 50:50 blend of 26 and 32 wt% loaded MPs in the presence of 0, 10 and 100 mM AAPH over 24 h (mean ± SEM, *n* = 3).

The absorbance of a 50 μg/ml curcumin solution in 0.1% (w/v) Tween 80 PBS (pH 7.4) was monitored to evaluate curcumin stability at 37°C (Fig. 2A). As shown, there was no significant change in absorbance, as well as no precipitation in solution, suggesting that curcumin was stable for over 24 h in aqueous solution. However, when in the presence of AAPH, curcumin’s absorbance profile dramatically changes over time. As free radicals are generated in solution, curcumin protons are abstracted leading to destabilization of the molecule and degradation of curcumin. As curcumin degrades, it loses its characteristic absorbance at 420 nm (Fig. 2A). The rate of curcumin consumption is correlated to the initial concentration of AAPH in solution. 10 mM AAPH solution results in 90% of curcumin consumed within the first 8 h, whereas 100 mM AAPH solution results in 90% of curcumin consumed within the first hour.

**Figure 3:**
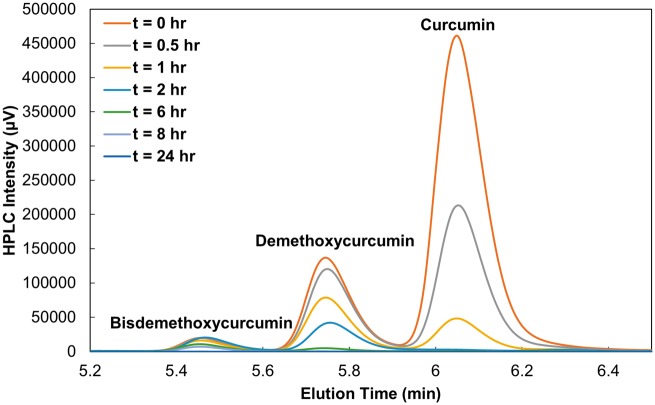
Curcuminoid consumption by AAPH over time.

Using HPLC, the consumption of the curcuminoids was investigated. The three forms of curcumin (curcumin, demethoxycurcumin and bisdemethoxycurcumin) found in solution were consumed in order of antioxidant activity [24], being that the curcumin peak diminished at a faster rate than demethoxycurcumin and bisdemethoxycurcumin (Fig. 3). Overall, curcumin appears to be highly susceptible to depletion in AAPH solutions at all times as its hydroxyl sites on each curcuminoid are always exposed.

### Microparticle degradation profiles in the presence of AAPH

26 wt% curcumin-loaded PBAE MPs were introduced to both 10 and 100 mM AAPH 0.1% (w/v) Tween 80 PBS solutions (pH 7.4) in a 37°C agitating shaker bath (Fig. 2B). The profile of the MP release in the presence of AAPH changed significantly compared with the release profile with 0 mM AAPH. Incorporating curcumin in to the backbone of PBAE crosslinked network provided protection from oxidation until released from the network. As the network hydrolytically degrades and releases intact curcumin, its exposed protons are able to be abstracted from the free radicals present. As curcumin is released, it is also consumed at a rate in which it is interacting with the free radical. Once the curcumin is released in its entirety and the MPs are fully degraded after 8 h, the residual curcumin released into solution begins to be consumed; however, the rate of consumption appears to be slower compared with free curcumin and after 24 h there is still is curcumin present based on absorbance. The controlled release of curcumin allowed for sustained concentrations of curcumin over an extended period compared with the exponential consumption seen of curcumin as a free drug. A total of 32 wt% curcumin-loaded PBAE MP was also introduced to AAPH (Fig. 2C). Initially less curcumin is recovered in the system, due to its slower release rate. The profile shifts its maximum amount of curcumin in solution with AAPH, where 40% of the absorbance is retained from the first 8 h in the 26 wt% MP to 12 h in the 32 wt% MP system. Again, after all of the curcumin has been released, the absorbance starts to decrease as the free curcumin in solution is consumed, but likewise to the other system, 10% of the absorbance remains even after 24 h. Similar trends are seen at high concentrations of AAPH for both 26 and 32 wt% MP networks; however the peak absorbance recovery is shifted by 2 h, the overall recovery is lower, and all of the colorimetric products are consumed by 24 h. For 100 mM AAPH, no color absorbance is seen after 12 and 20 h, respectively. To try and extend the curcumin in solution for a constituent amount of time, the 50:50 MP blend was evaluated as well (Fig. 2D). Within the first 8 h, 42% of recovery is obtained from the MP system, and due to the extended release contribution from the 32 wt% MPs in solution, the recovery at 40% was held constant between 8 and 10 h, before a decrease in recovery was seen. Similarly, the blend in the presence of the 100 mM AAPH was consumed at a faster rate, but also had a constant recovery of 15% for 2 h before it began to be consumed over the remaining 18 h. When observing free curcumin in the presence of 10 and 100 mM AAPH, the amount of curcumin decreases to 20% after 4 h and 20 min, respectively. For the controlled release mechanisms, the curcumin has sustained recovery above 20% for up to 16 h out of the 24-h duration. For the 100 mM AAPH solution, the recovery of curcumin is highest in 26 wt% as the rate of release of curcumin dominates the generation of radicals that would consume the active component over time. The slower release provides a slow enough rate to allow for a consistent consumption of curcumin over time, where curcumin recovery levels are low throughout the entire duration of release.

**Figure 4:**
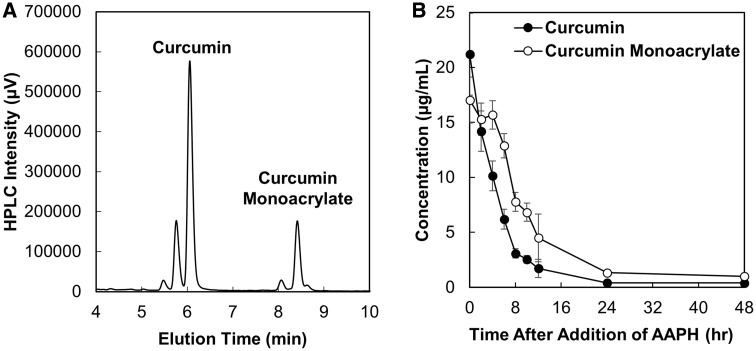
HPLC chromatogram of the curcumin and curcumin monoacrylate peaks found in the supernatant of a fully degraded MP system (**A**) and the independent consumption profiles of curcumin and curcumin monoacrylate in the presence of 10 mM AAPH (**B**) (mean ± SEM, *n* = 3).

Due to the residual absorbance retained at 24 h in all release curves interacting with 10 mM AAPH (Fig. 2B–D) and the change in extent of consumption after the MPs have been fully degraded, HPLC analysis was used to verify the degradation products present after the MP system had been completely degraded. After running the supernatant of a fully degraded MP system via HPLC, all three forms of curcumin (curcumin, demethoxycurcumin and bisdemethoxycurcumin) peaks were observed in Fig. 4A at an elution time of 5–6.5 min; however, residual curcumin monoacrylate peaks at elution times between 8 and 9 min were observed as well, verified by a CMA standard. The residual acrylates found in the supernatant could be a result of a curcumin triacrylate group being unable to fully cross-linked at all three sites due to steric hindrance of the curcumin molecule during film synthesis. This would allow for hydrolysis to retain two phenol groups but leave a residual unreacted acrylate group on the molecule. The additional acrylate moiety found on the molecule appeared to influence susceptibility of consumption, explaining the residual color left in solution over 24 h and the change in overall rate of consumption.

In total 26 wt% MPs were fully degraded in a 0.1% (w/v) Tween 80 PBS solution (pH 7.4). 10 mM AAPH was introduced to the solution with the released products, and the consumption profiles of curcumin and curcumin monoacrylate were monitored using HPLC coupled with UV-Vis (Fig. 4B). Curcumin was consumed over time at a consistent rate as seen before as a free molecule (Fig. 2A); however, curcumin monoacrylate peaks in the chromatogram do not begin to decrease until 4 h after the addition of 10 mM AAPH and then begin to be consumed. This lag could be due to decrease in antioxidant activity with the presence of the acrylate group on close to 40% of the curcumin molecules that are released; however, it was able to possess activity and was still consumed over time. There is also residual concentration of curcumin monoacrylate observed at 24 h.

### Development of the oxidative consumption rate model of free curcumin

**Figure 5:**
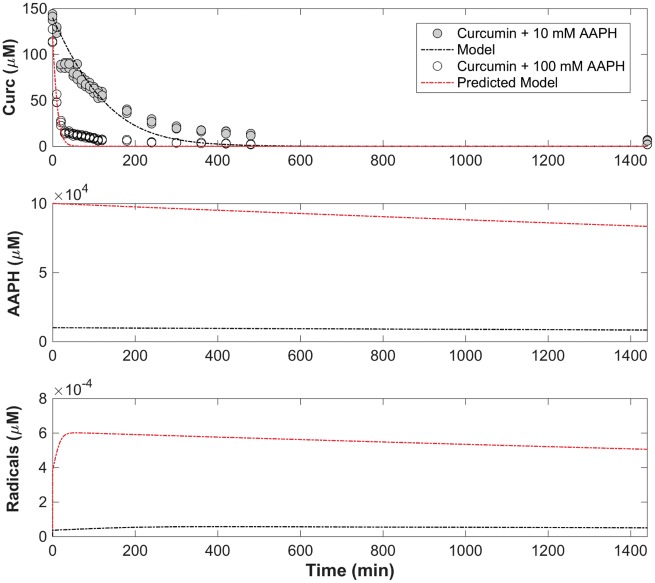
Model for curcumin consumption minimized based on raw experimental data of degradation products in the presence of 10 mM AAPH and verified by overlaying the predicted model with raw experimental data at 100 mM AAPH concentration.

Using the experimental data obtained from curcumin in the presence of 10 mM AAPH (Fig. 2A), Equations (2, 3a and 4) were solved simultaneously using MATLAB ODE15s solver and the fminsearch function to find the unknown consumption parameter, *k*_C_, to be 200 µM^−1^min^−1^ with *R*^2^ = 0.895 in comparison to the experimental data (Fig. 5). Equation (4) is plotted in the first subplot, where Equations (2 and 3a) are plotted in the second and third subplot. The model was verified by plotting the expected profile of curcumin consumption in a 100 mM AAPH solution and was compared with the experimental data of curcumin consumption in 100 mM AAPH over time. The consumption profile of curcumin exponentially decreased, and the entirety of curcumin was theoretically consumed within the first hour. Although the finality of curcumin consumption was not until 8 h experimentally, the majority of curcumin was consumed within the first hour, just as the model described. The consistency in predicted curcumin consumption profile verified second order degradation kinetics to describe the consumption of curcumin in the presence of AAPH. Correlation factor values are provided in Table 1. AAPH concentration decreased over time as the thermal decomposition of the molecule takes places. The concentration of free radicals started to increase as the free radicals were generated and then began to decrease as the elimination constant within the equation dominated after all the curcumin was consumed. This model was able to predict the consumption profile of curcumin over time in a solution of higher radical concentration at a correlation factor of 0.915.

**Table 1:** Oxidative consumption rates and correlation factors for the minimized and predicted curcumin consumption profile

Model	*k*_c_ (μM^−1^ min^−1^)	*R* ^2^
Curcumin + 10 mM AAPH	200	0.895
Curcumin + 100 mM AAPH	200	0.915

### Modeling the consumption of released products in the presence of AAPH

**Figure 6:**
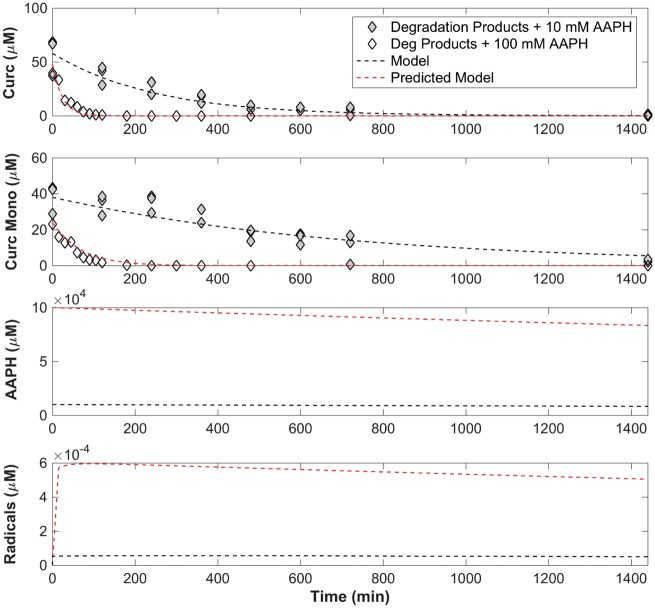
Model minimized based on experimental data of the curcumin and curcumin monoacrylate release products in the presence of 10 mM AAPH and verified by overlaying predicted model with experimental data at 100 mM AAPH concentration.

Equation (5) was added to the set of equations to describe the consumption rate of the curcumin monoacrylate released. Equations (2, 3b, 4 and 5) were solved simultaneously as previously described and k_C_ and k_CM_ were minimized fitting the experimental results to 75 and 25 µM^−1^min^−1^_,_ respectively (Fig. 6). It is here we note that the oxidative consumption rate of curcumin has decreased in comparison to the oxidative consumption rate of curcumin as a free molecule. This suggests the activity of curcumin was slightly inhibited in the presence of residual degradation products from the polymer network in solution or from being integrated within the network. The model was then used with the minimized parameters to predict consumption profiles of curcumin and curcumin monoacrylate in the presence of 100 mM AAPH and overlaid with experimental data. Indeed, the rate parameter of curcumin monoacrylate was lower; however, when predicting consumption profiles in a higher concentration of free radicals, the model fit well at a correlation factor of 0.924, again providing validity to the second order kinetic rate model for the products released from the network (Table 2).

**Table 2:** Oxidative consumption rates of curcumin and curcumin monoacrylate release from MP networks

Model	*k*_C_ (μM^−1^ min^−1^)	*k*_CM_ (μM^−1^ min^−1^)	*R* ^2^
Degradation Products + 10 mM AAPH	75	25	0.876
Degradation Products + 100 mM AAPH	75	25	0.924

The free radical levels present over time in this model (Fig. 6, subplot 3) appeared to be more constant than in the curcumin model with a low but instantaneous production of free radicals, thus decreasing slightly over time due to the elimination rate term. Within both the curcumin and release product models (Figs 5 and 6), the fit appears to deteriorate slightly over time. This could be due to the *k*_c_ and *C*_c_ terms describing curcumin as an overall component. The starting material used was a mixture of three curcuminoids with curcumin the major component of the mixture (85%). In Fig. 3, curcumin has the fastest consumption rate compared with demethoxycurcumin and bisdemethoxycurcumin. Because these three compounds were described as one consumption rate, the relative consumption will be skewed to the most active form, and therefore model a faster consumption profile overall.

For the treatment of an oxidative stress-induced disease such as OM, the application of curcumin conjugated PBAE MPs to the surface of the buccal cheek pouch could allow for controlled release of curcumin to suppress free radical production that activates pro-inflammatory markers [25]. When translating this model into human or *in vivo* studies, other pathways of elimination of curcumin may be added. A potential missing term or ‘piece to the puzzle’ would be a rate to describe the wash out of particles, which would account for any loss of curcumin that was removed before release. This would take in to account the retention time of particles on the surface of the buccal tissue. Retention time can be enhanced by using a vehicle such as a mucoadhesive solution, but this term would and should be incorporated in future development of models to predict lasting effects *in vivo.*

Another factor that could play a role in the discrepancy of the consumption profiles is the rate of hydrolysis versus rate of swelling. Although PBAE crosslinked networks are degradable, they are also classified as hydrogels due to their swelling properties [16, 26]. Swelling effects can be demonstrated in bulk films, where in each independent network, there is a point at which network integrity is lost, allowing for the remainder of the curcumin to be released; however, this effect is not as prominent in MP systems as the increase in surface area promotes constant release compared with bulk films.

The model developed demonstrates the ability to describe curcumin and curcumin monoacrylate in the presence of a free radical, like AAPH, and has promising results to better understand free radical interaction of curcumin *in vivo.* The consumption rate of curcumin and curcumin monoacrylate in the presence of AAPH was successfully described by this nonlinear model; however, consumption rates of these compounds could change based on the origin and property of the free radical present. It has been shown that the activity of scavenging potential changes based on the interaction of radicals. Curcumin has been presented as having a high reducing power to transition metals, a value correlated to antioxidant capacity, but when investigated with specific free radical interactions, lower scavenging potentials are found for superoxide anions and H_2_O_2_ compared with AAPH [27]. In the future, this model could be investigated and compared against consumption profiles of other free radical molecules to better understand the degree of translation in other environments and the authenticity this model has to AAPH specifically.

### Demonstration of controlled release antioxidant delivery

**Figure 7:**
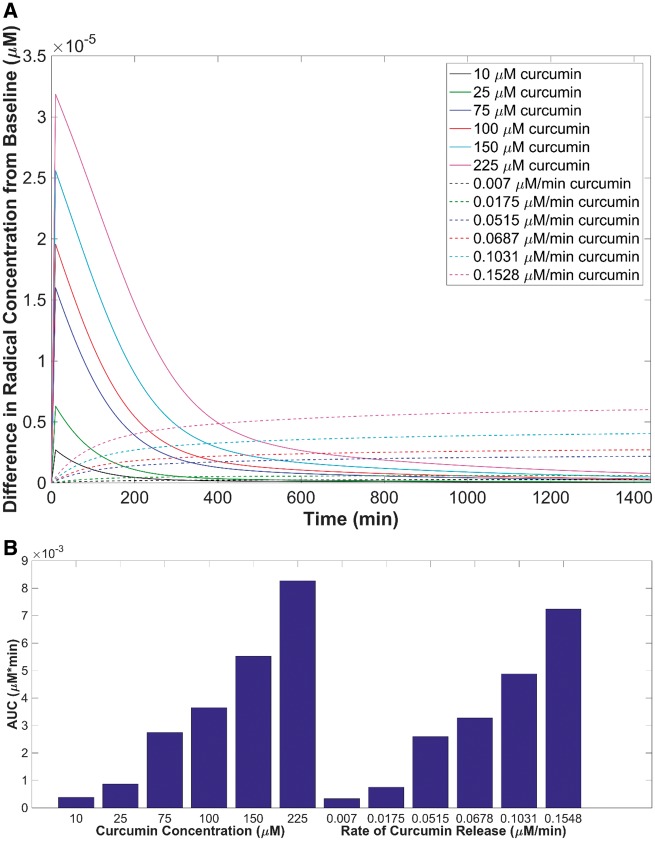
The deviation of free radical concentration when the addition of a one-time theoretical delivery of free curcumin is added to a 10 mM AAPH solution or the rate equivalent to the total amount added over 24 h is introduced (**A**) and the AUC values (µM*min) reported as a bar graph (**B**).

The deviation of free radical concentrations by adding curcumin at a constant rate or at one initial amount was compared. The theoretical baseline generated at a concentration of 10 mM AAPH was the point of deviation. The plot shown in Fig. 7A represents the concentration deviation from the baseline concentration of free radicals over time in the presence of bolus curcumin doses or constant release rates of equivalent curcumin over 24 h, where *C*(*t* = 0) = 0 μM. For initial curcumin concentrations of 10 and 25 µM, the change in free radical concentration was 0.25 × 10^−5^ and 0.6 × 10^−5^ µM initially and reverted to baseline within <2 h after all the curcumin had been consumed. Bolus delivery of low concentrations allowed for a fast, initial consumption of radicals present, and deviated significantly from the baseline level of radical concentration. The curcumin was then quickly consumed, and the initial free radical levels were maintained. Higher initial concentrations such as 225 µM curcumin deviated significantly at *t* = 0 min by 3.25 × 10^−5^ µM and did not reach baseline until after 24 h. The greater the initial dose of curcumin, the longer it took for the free radical concentration to reach baseline, which initially could appear as a benefit; however, the greater the deviation in antioxidant/oxidant levels, the higher the risk in implementing imbalance to the cellular environment. This could ultimately create an antioxidant toxicity effect by shifting the redox equilibrium in the environment. This model represents the potential risk of toxicity by showing the drastic change in concentration initially.

When utilizing the model with curcumin at a constant rate of release over 24 h, the deviation of free radical concentration did not appear as significant compared with the bolus dose delivery method. Unlike bolus doses of curcumin, the controlled release of even the highest theoretical delivery of 225 µM over 24 h only deviated from baseline levels by 0.6 × 10^−5^ µM at any time observed in the 24-h period. To understand the degree of curcumin’s scavenging potential and the degree in change of the free radical environment, the area under the curve (AUC) for each delivery method was evaluated (Fig. 7B). For the curcumin rates of release, the AUC ranged from 3.34 × 10^−4^ to 7.3 × 10^−3^ µM*min, and for initial doses of curcumin, the AUC ranged from 3.84 × 10^−4^ to 8.3 × 10^−3^ µM*min. For all bolus deliveries, the AUC was higher than all the controlled release models, showing greater change in antioxidant/oxidant levels in bolus deliveries for each theoretical dose equivalence (Table 3).

**Table 3:** Summary of the total AUC of each theoretical dose

Initial dose (μM)	AUC (μM*min)	Rate of release (μM/min)	AUC (μM*min)
10	3.84 × 10^−4^	0.0070	3.34 × 10^−4^
25	8.72 × 10^−4^	0.0175	7.56 × 10^−4^
75	2.7 × 10^−3^	0.0515	2.6 × 10^−3^
100	3.7 × 10^−3^	0.0678	3.3 × 10^−3^
150	5.5 × 10^−3^	0.1031	4.9 × 10^−3^
225	8.3 × 10^−3^	0.1548	7.2 × 10^−3^

## Conclusions

In these novel controlled delivery systems, the consumption of curcumin was protected as it was incorporated into the backbone of the MP network until hydrolyzed and released into the environment. The experimental findings in this work showcased the ability to deliver a constituent amount of curcumin over time through a controlled release system compared with rapid consumption as a free drug. Experimental data provided a foundation to develop a second order kinetic rate model to describe the oxidative consumption of curcumin as a free molecule and the oxidative consumption of the materials released from the MP network utilizing a first principles process. This gives insight on the incorporation of an antioxidant into the backbone of a polymer and how the addition of curcumin into a network allows for more consistent delivery and protection of curcumin over time. It also showed that controlled release can suppress levels of free radicals theoretically over time consistently rather than dramatically, which is not possible with bolus delivery of curcumin. This model will continue to be developed to advance the pharmacokinetics of curcumin conjugated PBAE networks and implement them into clinical practice in the future.
