# Corrigendum to: Modeling the oxidative consumption of curcumin from controlled released poly(beta-amino ester) microparticles in the presence of a free radical generating system

**DOI:** 10.1093/rb/rbz047

**Published:** 2019-12-27

**Authors:** Carolyn T Jordan, J Zach Hilt, Thomas D Dziubla

**Affiliations:** Department of Chemical and Materials Engineering, University of Kentucky, Lexington, KY 40506, USA

doi: 10.1093/rb/rbz002, *Regenerative Biomaterials* 2019;6:201–210

The original version of this article incorrectly declared no conflicts of interest. The correct conflict of interest statement on behalf of the author has now been included.

